# Spontaneous hemoperitoneum in the second and third trimester of pregnancy: two uncommon case reports at Tu Du Hospital, in Vietnam and a literature review

**DOI:** 10.1186/s12245-023-00498-w

**Published:** 2023-04-17

**Authors:** Anh Dinh Bao Vuong, Thanh Hai Pham, Xuan Trang Nguyen, Ngoc Bich Trinh, Phuc Nhon Nguyen, Quang Nhat Ho

**Affiliations:** 1 Department of High-Risk Pregnancy, Tu Du Hospital, Ho Chi Minh City, Vietnam; 2 Tu Du Clinical Research Unit (TD-CRU), Ho Chi Minh City, Vietnam; 3 Department of Postoperative Care, Block A, Tu Du Hospital, Ho Chi Minh City, Vietnam

**Keywords:** Emergency, Hemoglobin, Maternal mortality, Pregnancy, Fetal death, Spontaneous hemoperitoneum, Ultrasound

## Abstract

**Background:**

Spontaneous hemoperitoneum in pregnancy (SHiP) refers to fluid collection in the abdominal cavity with a vague presentation of clinical symptoms. Particularly, SHiP causes a life-threatening condition with the coexistence of intrauterine pregnancy, since this dangerous complication significantly increases the maternal and fetal mortality. Herein, we present two cases of nontraumatic SHiP in the second and third trimester of pregnancy, respectively.

**Case presentation:**

The pregnant woman in case 1 was admitted to our hospital owing to severe paroxysmal shoulder pain along with abdominal pain. Her medical history was remarkably recorded with endometriosis and adenomyosis. At the emergency room, an ultrasound scan revealed a live fetus corresponding to 21 weeks and 3 days and free fluid in the abdominal cavity. She was subsequently diagnosed with SHiP and underwent immediate laparotomy for hemostatic procedures. During the postpartum course, the patient was uneventfully monitored. Unfortunately, the patient delivered on the 4th postoperative day in spite of the initial administration of tocolytic agents and close monitoring. The primigravid woman in case 2 complained of lower abdominal pain and vaginal bleeding. The patient’s history was noted with ovarian tumor removal. At admission, the sonography scan revealed free fluid in the abdominal cavity, a fetus at 34 weeks and 3 days gestational age with bradycardia of 70 bpm, and a laboratory test showed a low hemoglobin level. Thus, exploratory laparotomy and hysterotomy were performed at the same time due to fetal distress. The postpartum course was uneventful. The patient was discharged 5 days later.

**Conclusions:**

In pregnant women with a history of endometriosis, adenomyosis, or ovarian tumor removal, acute abdominal pain combined with the presence of free fluid collection in the intraperitoneal cavity, and a decreased hemoglobin levels should be first assessed as SHiP originating from the spontaneous rupture of abnormal vascular proliferation. Proper management is strongly indicated for an emergent laparotomy to control the active bleeding point, thus increasing the survival rate for both mother and neonate.

**Supplementary Information:**

The online version contains supplementary material available at 10.1186/s12245-023-00498-w.

## Background

Spontaneous intraperitoneal hemorrhage or abdominal apoplexy is a very rare uncommon occurrence in connection with an intrauterine pregnancy. In general, the etiologies are diverse and always difficult to determine before surgery. Yang et al. reported four cases of SHiP, including spontaneous rupture of the uterine veins, spontaneous rupture of the liver, rupture of the external iliac vessel branch, and rupture of the right renal hamartoma [1]. Meanwhile, Vuong et al. revealed the unscarred uterine rupture due to placenta accreta spectrum led to severe SHiP [2]. Xu et al. also described 3 cases of intraabdominal bleeding with the different causes [3]. In particular, some authors have recently reported a massive hemoperitoneum caused by endometriosis eroding into the branch of the uterine artery [4–6].

Accordingly, an accurate diagnosis of SHiP should be based on clinical evaluation accompanied by imaging modalities. Mostly, delayed detection leads to catastrophic presentation of symptoms, resulting in maternal hypovolemic shock, fetal distress and high rate of fetal death [7]. In cosistent with the report of Lier et al., the perinatal mortality rate was 26.9% (18/67 fetus) [8]. In the interim, timely and multidisciplinary management was the pivotal point to reduce perinatal mortality during pregnancy and puerperium [9].

We hereby report two uncommon cases of SHiP to emphasize the clinical features, characteristics of ultrasound scan, and increase the appropriate awareness of physicians.

## Case presentation

### Case 1

A 39-year-old primigravida pregnant woman was hospitalized at the local hospital for paroxysmal shoulder pain on the right side. Subsequently, the patient was transferred to our hospital due to free fluid collection and hydronephrosis. Eight years ago, the patient’s antecedent was recorded with adenomyosis and endometriosis tumor on the bilateral ovaries without treatment. She denied having trauma, coitus, and straining on the day prior to hospitalization. She conceived naturally after 3 years of marriage. On the early morning of the same day before admission, the patient was complained of right upper quadrant pain onset, that extended to the thigh, and the shoulder on the same side. She woke up in the middle night because of intensive paroxysmal pain, and could not even be mobile. At admission, a remarkable tachycardia was noted at 110 bpm, her blood pressure was 140/100 mmHg, respiratory frequencies were 20 times/min, and her body temperature was 36.5 degrees. The shock index based on the heart rate/systolic BP was calculated at 0.8 (greater than reference range from 0.5 to 0.7). Abdominal palpation indicated generalized tenderness.

**Fig. 1:**
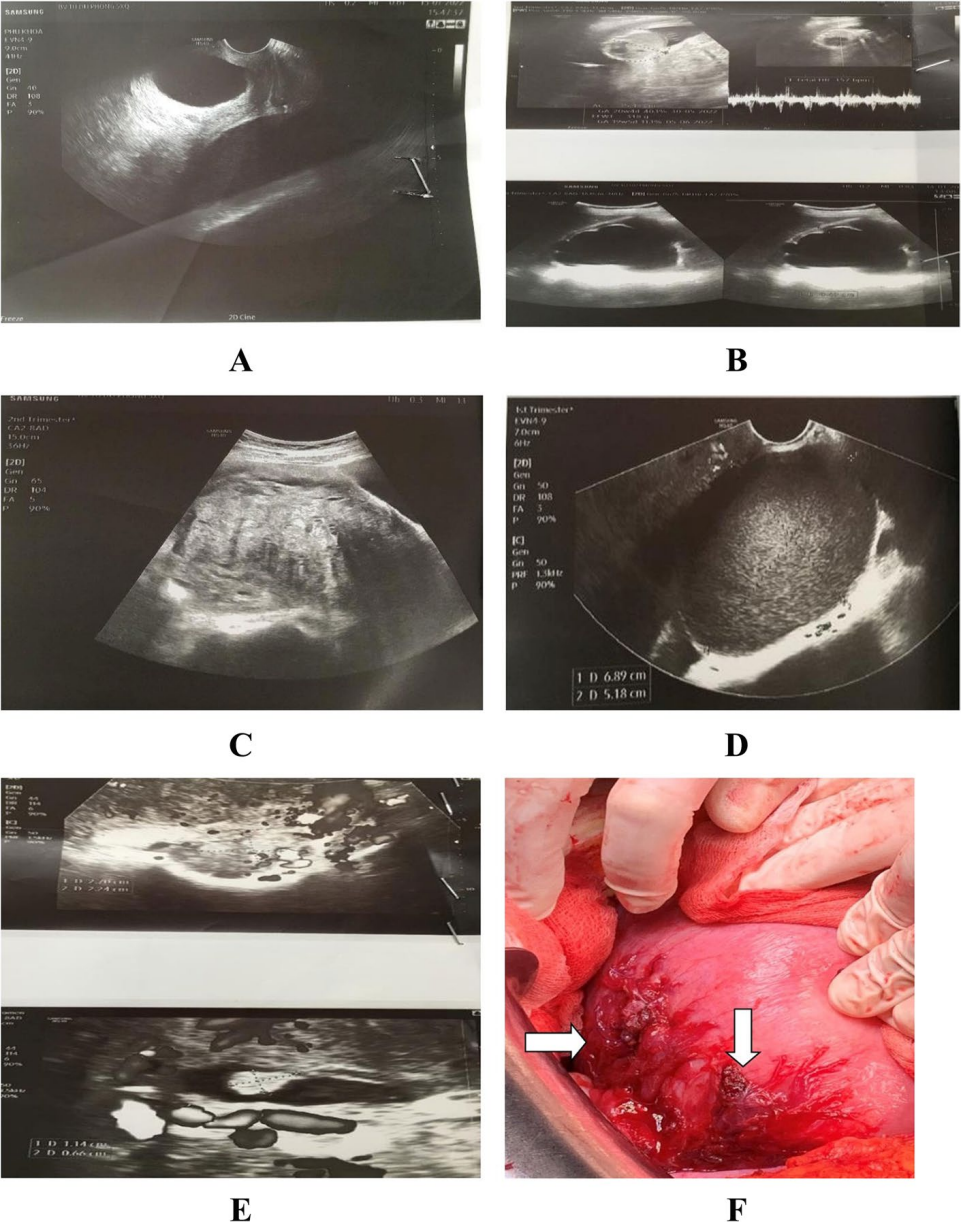
Ultrasound scan shows as follows: **A** free fluid in the pelvic cavity. **B** Single alive fetus at 21 weeks and 3 days of gestation and maternal hydronephrosis at the third grade. **C** Adenomyosis image. **D** Endometriotic cyst. **E** Hypervascularity on the lateral wall of the uterus. **F** Abnormal appearance with laceration on the serosal surface of the uterus and vessel ligations (white arrow) were performed during exploratory laparotomy

On the obstetric examination, the uterine height was measured at 19 cm, the fetal heart rate was 145 bpm, and uterine contraction was absent. The cervix was closed and vaginal bleeding was not present. The amniotic membrane was intact. Conversely, an ultrasound scan revealed a single a live fetus corresponding to 20 weeks and 3 days and abdominal fluid consistent with hemorrhagic fluid was recorded with a large amount. The depth of the heterogenous fluid pocket was bilaterally measured at 74 mm in the right iliac fosse and 80 mm in the left iliac fosse. Images of adenomyosis and the right endometriotic cyst were found. Hydronephrosis of the third degree was also observed (Fig. 1). Laboratory examination findings were as follows: low hemoglobin level at 8.6 g/dl, hematocrit of 24.6%, white blood cells of 16.29 cells/mm^3^, and platelets of 276 cells/mm^3^. Coagulopathy was normal. Tumor markers were revealed with cancer antigen-125 (CA-125) of 58.8 UI/ml (reference value ≤ 35), human epididymal protein-4 (HE-4) of 74.0 pmol/l (reference value ≤ 70), risk of ovarian malignancy algorithm (ROMA) value of 18.22% (reference value ≤ 25.3), and alpha fetoprotein (AFP) of 111 ng/ml (reference value < 7). The urinalysis test was normal.

The patient was immediately indicated for an emergency laparotomy with vertical skin incision owing to intraabdominal hemorrhage. Upon laparotomy, the abdominal cavity was filled with a large amount of fresh liquid and clotted blood. After suctioning the blood, we explored the entire abdominal cavity to identify the source of bleeding. No of ovarian tumors were found. The right ovary was adherent to the uterine body. The vasculature was abnormally proliferative at the right corner of the uterus. The uterus was in the form of adenomyosis. To stop bleeding, the hemostatic suture was carefully performed. In addition, an absorbable agent such as gelatin sponge was added to the hemostatic position. Then, abdominal drain was placed on the left iliac fossa. Intraoperatively, total blood loss was 2000 ml. The uterus was preserved. The patient was administered 2 units (350 ml/unit) of packed red blood cells (RBCs) in the operating room. The team had no specimens for histopathological assessment.

Postoperatively, the infectious bilan was revealed with increased white blood cells up to 19.45 cells/mm^3^ and CRP of 117.3 mg/l. The urine culture was negative. The patient was treated with broad-spectrum antibiotics including Tazocin every 8 h, which was then replaced by Meropenem every 12 h. The hemoglobin level was 6.9 mg/dl. Thus, the patient received an additional 2 units of packed RBCs. Due to the high risk of miscarriage, tocolytic agents were given with tractocil therapy and continuation of nifedipine. However, uterine contraction and cervical dilation were regularly progressive; thus, spontaneous labor could not be avoided. Consequently, the patient was delivered with a baby of 21 weeks of GA on the fourth postoperative day. The patient was released from the hospital on the 3rd day of postpartum and was sent to a nephrologist for the management of hydronephrosis.

### Case 2

A 33-year-old pregnant woman (G0P0) was hospitalized at our tertiary referral hospital due to complaints of lower abdominal pain and vaginal bleeding on arrival. It was not associated with nausea, vomiting, fever, headache, or blurring of vision. Her obstetrical history revealed with no complication except for an arabin cervical pessary, and one abortion. Her medical record was unremarkable except for the right ovarian tumor resection. Physical examination findings were as follows: blood pressure of 100/60 mmHg, pulse rate of 86 bpm, and the body temperature of 37° celcius. The patient’s skin was pale. During hospitalization, her symptoms deteriorated with the shock index was calculated at 0.9. At the obstetrical examination, the uterus size corresponded with the gestational age at 34 weeks and 3 days. The cervical dilation was about 1 cm and cervical motion tenderness with bulging posterior fornix. At the speculum, the blood clot was observed. There was diffuse direct and rebound abdominal tenderness.

**Fig. 2:**
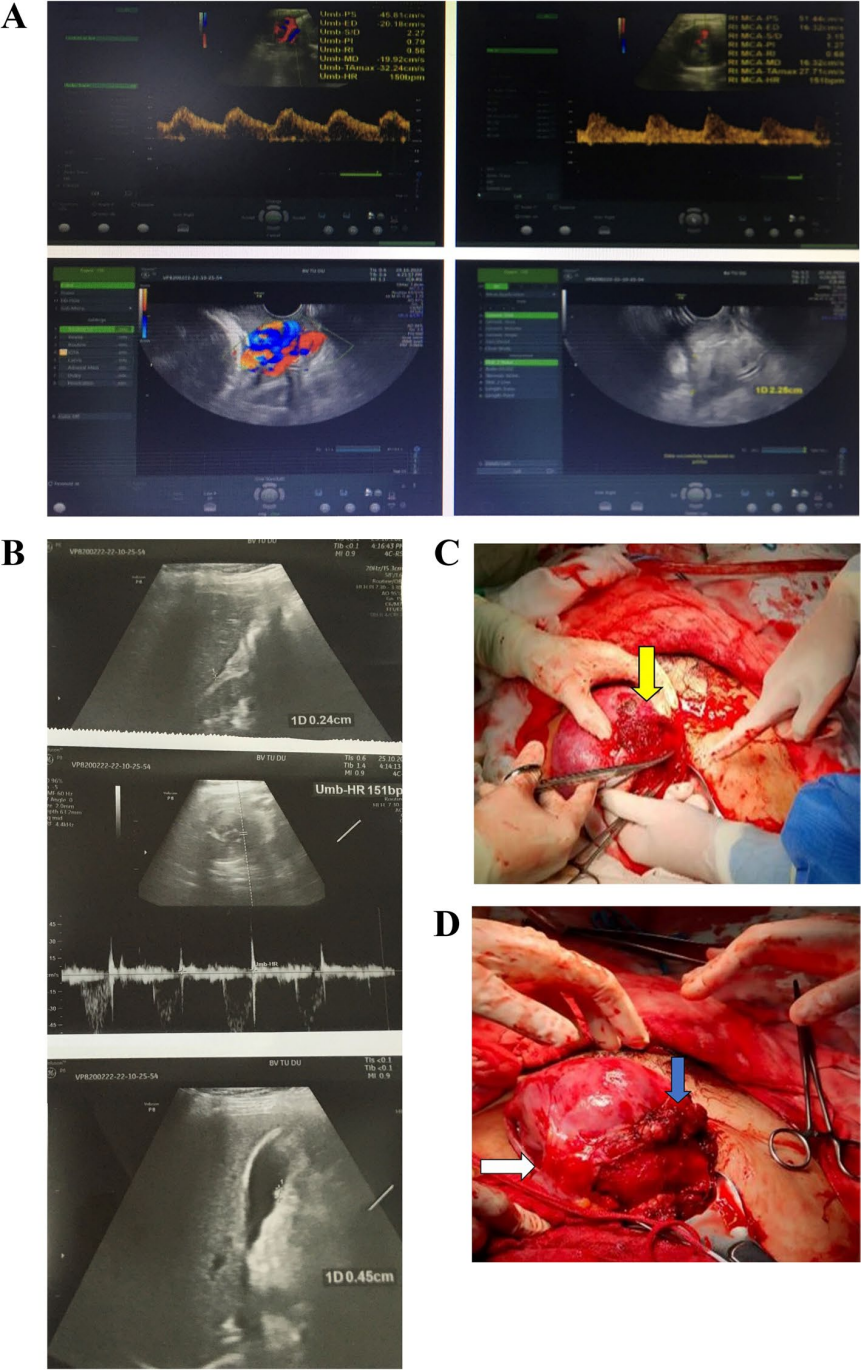
Ultrasonography shows **A** a single a live fetus and proliferative vasculature at the cervix. **B** Free fluid collection in the abdominal cavity. **C** Extravasation in the right adnexa, suspected to arise from the right utero-ovarian plexus (yellow arrow). **D** Uterine closure accompanied with a multiple hemostatic sutures were performed (blue arrow). One of the adherent bandages existed between the lateral posterior of the uterus and the abdominal anterior wall of the abdomen (white arrow). The bleeding stopped after releasing a part of the adhesion, excision of fragile tissue, and suturing

At admission, a transabdominal ultrasound scan revealed an alive fetus at the cephalic presentation and a decreased of the pulsatility index of middle cerebral artery at the percentile of 1%. The lower margin of the placenta was located nearly the internal os cervix, thus, suspicion of low-lying placenta was made, and free fluid collection was observed. The heterogenous fluid layer was measured at thickness of 29 mm in the right iliac foss and 12 mm in the left side. There was a small amount of free fluid in the perihepatic and perirenal space. Bilateral ovaries were not found. Small polyp measured 4–5 mm in the gallbladder. On ultrasound, fetal bradycardia was down to 70–100 beats/min without signs of placental abruption. Immediately, an abdominal ultrasound scan by transvaginal and transabdominal probe was carefully performed. The turbulent hypervascularity was revealed at the cervix and uterine surface (Fig. 2). In addition, fluid collection increased to 37 mm in maximum measurement without an identifiable origin. Fetal cardiotocogram could not be performed because of the urgent situation. Additionally, irregular uterine contraction was approximately one per minute. After consultation, the pregnant woman was requested for emergency caesarean delivery under general anesthesia due to fetal hypoxia and suspicion of intra-abdominal hemorrhage.

Upon entering the abdomen with vertical skin incision, a massive hemorrhage and active bleeding point were found predominantly along the parametrial region with a distorted and friable appearance of the right adnexal structure. Particularly, existing adherent fibrous structure from the anterior and posterior surface of the uterus to the abdominal wall. Initially, owing to suspicion of fetal distress, the patient was subjected to a low transverse isthmic incision. A live male baby weighing 2200 g was extracted with Apgar scores of 6 and 7 at 1 and 5 min, respectively. The placenta was intact and delivered manually. Following uterine closure, at the right cornus of the uterus involving the utero-ovarian plexus, a hematoma 3 × 4 cm in size was found with active bleeding vessel during exploration. In addition, the great omentum, bowel, and fibrous band were adherent to the ovarian artery and the right lateral wall of the uterus. The abdominal incision was widened. Due to the obstructed view by the gravid uterus, uterine exteriorization was carried out. Then, adhesion was released, electrocoagulation was used and the static sutures were inserted to control the bleeding point on the superficial uterine surface (Supplementary video 1 A, B). Following abdominal examination, the liver region and splenic area were normal. A pelvic drainage was placed after hemostasis was ensured. In total, estimated blood loss was 1200 ml. Two units of whole blood were cross-matched initially and were transfused during surgery. The team had no removed tissue for histopathological examination.

In the intensive care unit, the vital signs were stable with a blood pressure of 110/70 mmHg, a body temperature of 37°, and a pulse rate of 86 bpm. Drain was recorded at 50 ml fluid in brownish color. The coagulopathy profile was normal. During the postpartum course, the patient was administered broad-spectrum antibiotics due to the high level of white blood cells of 20.41 cells/mm^3^, which then decreased gradually to the normal limit. The patient recovered uneventfully and was discharged with satisfactory feelings 5 days later. The hemoglobin level increased from 10.0 to 10.6 g/dl. A routine 4-week follow-up appointment at the outpatient gynecological clinic was uneventful.

## Discussion

During pregnancy, the coexistence of intrauterine fetus and SHiP is rare. In early pregnancy, the etiology of extrauterine pregnancy or heterotopic pregnancy may occur. However, in the second and third trimester, the cause of abnormal vascular ruptures was more common [3]. Despite its rarity, rupture of proliferative vessels in the patient with a history of endometriosis and adenomyosis during pregnancy has been mentioned in the literature. Thus, endometriosis and adenomyosis may be etiologies in this entity. These benign gynecologic pathologies cause a chronic inflammatory process on the uterine surface, resulting in abnormal proliferative vasculature. Moreover, during pregnancy, the blood flow via the uterus increases, and the proliferative vessels become hypertrophic and fragile. Along with or without triggering factors changing the abdominal pressure, a sudden rupture of dilated subserosal vessels originating from endometriotic implants causes SHiP [10–12].

In 2020, Kim et al., mentioned endometriosis induced massive hemoperitoneum which was misdiagnosed with the ruptured ectopic pregnancy [13]. In our first case, the patient had a history of bilateral ovarian endometriosis tumors before pregnancy and a right ovarian cyst of endometriosis as well as an adenomyosis lesion on prenatal US which was confirmed in laparotomy. This occurrence was in line with the study of Lier et al., who recognized that endometriosis was present in 33/59 cases (55.9%), most often diagnosed prior to pregnancy in a literature review [8]. In the second case, adhesions originating from the history of ovarian tumor removal could have spontaneously caused avulsion of the abnormal proliferation of utero-ovarian plexus. The connective band was observed during surgery. Previously, this report was rarely reported.

Regarding diagnosis, an initial confirmation of abruptio placenta or ruptured uterus should be excluded in the late term pregnancy with acute abdominal pain. Moreover, the differential diagnosis of a suddenly abdominal pain with hemodynamic collapse and no external bleeding included uterine rupture, sepsis, aortic dissection, and venous thromboembolism in pregnant women [14]. Accordingly, diagnosis of SHiP is more accessible with the presence of free fluid collection on ultrasound considering as the first line in detection. In addition, the main symptom is acute abdominal pain in almost 70% of patients combined with a low hemoglobin level. Hypovolemic shock is present in 18% of cases. Similar to the present case, in conditions of intraperitoneal hemorrhage not related to placental causes and the intensity of pain not related to the placental site but increasing when the patient is lying on the side, some patients could suffer shoulder tip pain in the supine position [15]. However, the symptoms of SHiP could also be overlapped and the diagnosis was made intraoperatively owing to caesarean section following fetal distress or other indications [3]. Furthermore, the symptoms may also be mimicked with acute peritonitis, thus a delayed surgical intervention made the situation worsen [12].

Importantly, posterior culdocentesis or puncture into the pouch of Douglas could be performed in the case of suspicion without strong evidence of SHiP, this procedure yielded non-coagulable blood in case of intraperitoneal hemorrhage [3]. Computed tomography and magnetic resonance imaging could also be necessary to aid the differential diagnosis, especially, these modalities can accurately reveal vascular lesions (aneurysm or pseudoaneurysm) [16]. Insufficiently, the limitation of our study is lacking of histopathological examination since the team could not remove any specimen during surgery.

Upon diagnosis, timely management with surgical intervention and resuscitation with blood transfusion must be taken into consideration the hemodynamic instability of the patient and gestational age of the fetus (Table 1). In the third trimester or the late term pregnancy, a surgical procedure with caesarean delivery should be indicated to reduce the mortality of the mother and the newborn [9]. In the second trimester, an intervention on uterus with superficial vascular proliferation increases dramatically the risk of miscarriage. Consequently, successful continuation of pregnancy remains low with only 7/45 cases (15.6%) reported according to Lier et al. [8].Therefore, in addition to the administration of tocolytic drugs, the risk of miscarriage should be informed for the patient. The recurrence of SHiP should also be vigilant during this pregnancy and for the next pregnancy [8, 17].

**Table 1:** Summary of spontaneous intraperitoneal hemorrhage related to the uterus and adnexa

Reports	GA, timing detection	Etiology, lesion position	Risk factors	Clinical symptoms and imaging modalities	Management	Outcomes
Sigurd et al. (1988) [7]	39 weeks	Blood oozing from a rupture of a uterine vein on the back side of the uterus	Not found	An intensive burning pain in the lower part of the abdomen and in the shoulders Deep hypotensive shock	Laparotomy for CS and hemostasis	Fetal death Maternal survival EBL at 3000 ml
Choobun et al. (2006) [17]	31 weeks	Spontaneous rupture of the utero-ovarian plexus	Not found	Acute lower abdominal pain, distended abdominal wall	Laparotomy for CS and sutured ligation	Maternal survival and alive newborn EBL at 750 ml
	24 weeks, then, recurrence at 31 weeks	Spontaneous rupture of the uterine varices	Not found	Acute abdominal pain, the right lower quadrant tenderness with guarding, and vomiting A moderate amount of free fluid in the peritoneal cavity on US	Twice laparotomy for hemostatic procedures	Vaginal delivery with alive newborn of 33 weeks GA EBL at 1500 ml and 2000 ml, respectively
Hamadeh et al. (2017) [15]	Third trimester	Ruptured vessels in the uterine–ovarian plexus	Not mentioned	Severe generalized abdominal pain	Laparotomy for immediate CS and hemostasis	Survival
Hardin et al. (2017) [16]	20 weeks	Spontaneous rupture of a uterine artery	Unknown	Suprapubic abdominal pain, emesis and vaginal spotting Decreased Hb of 9.7 g/dl and Hct of 28.9% Hb/Hct was 8.7 g/dl and 25.7% on follow-up	Emergent laparotomy surgery with ligation of active bleeding artery	The patient discharged at 22 weeks of GA and IOL at 36 weeks and delivered a 2346 g infant with Apgar scores of 8 and 9 via CS
Xu et al. (2019) [3]	40 weeks 6 days 3 h After vaginal birth	An area of 4 × 2 cm existed in the lower left posterior wall of uterus	Endometriosis	Paroxysmal pain, abdominal distention The Hb was from 10.31 to 7.48 g/dL. Abdominal CT and abdominal ultrasonography indicated presence of free fluid in the abdominal cavity	Open exploration and hemostasis	Survival EBL and blood transfusion were about 3600 ml and 1600 ml
	40 weeks 2 days	Multiple inflammatory adhesions and multiple active bleeding related to the rupture of endometriosis cyst. A local hematoma of 4 × 4 × 11 cm was found	Left ovarian cyst about 3 × 4 × 5 in size	Not reported SHiP was coincidentally found during CS due to fetal distress	CS and hemostasis	Survival EBL and blood transfusion were about 2000 ml and 800 ml, respectively
	25 weeks 5 days	The active bleeding was seen near the posterior lobe of broad ligament on the left posterior wall of uterus. Extensive hyperemia, edema, and inflammatory exudation were found in the surrounding tissues	History of laparoscopic surgery	Paroxysmal pain in lower abdomen Hb 7.3 g/dl Emergency abdominal ultrasonography and abdominal CT indicated large amounts of effusion in abdominal cavity. Noncoagulant blood was drawn out from the abdominal cavity	Open exploration and hemostasis	Fetal death EBL and blood transfusion were about 2400 ml and 2200 ml, respectively
Yang et al. (2020) [1]	29 weeks 1 day	Ruptured subserosal vein on the posterior uterine wall	Uterine malformation with didelphic uterus	Lower abdominal pain, nausea, dizziness, palpitations, and anal bloating Hb was 88 g/L, and Hct was 26% A large amount of fluid was seen in the pelvis and abdominal cavity on US Posterior culdocentesis yielded non-coagulable blood	Exploratory laparotomy, CS, and control of bleeding	Fetal death EBL at 1900 ml six units of packed RBC intraoperatively
Kim et al. (2020) [13]	First trimester	Continuous active bleeding was observed from the peritoneal wall of the pouch of Douglas	Endometriosis	Acute abdominal pain, vaginal bleeding, peritoneal irritation signs along with hemodynamic instability Decreased Hb to 7.0 g/dl Ultrasonographic evidence of pelvic fluid collection	Emergency exploratory laparoscopy and laparoscopic electrocoagulation	Survival EBL was 1800 ml Received 3 units of RBC transfusion Spontaneous abortion
Silva et al. (2020) [14]	22 weeks	A laceration of the left posterior leaf of the broad ligament. An active site of bleeding from the left uterine artery branch with blood pulsating	Not found	General malaise, worsening abdominal pain, and hemorrhagic shock Drop in Hb levels US revealed an echogenic image with 95 × 88 × 53 mm suggestive of a blood clot on the pouch of Douglas Immediate evaluation of the CT scan images revealed haemoperitoneum	Exploratory laparotomy and hemostasis	Survival EBL was 2000 mL. Intraoperatively, the patient was resuscitated with 1600 mL of crystalloids, 4 units of erythrocyte concentrate, 3 units of FFP, 2 g of fibrinogen, and 1 g of tranexamic acid Pulmonary thromboembolism on the 15th day of postoperation
Huang et al. (2021) [12]	18 weeks	Bleeding from decidualized endometriotic tissue over posterior uterine surface	Endometriosis	Diffuse lower abdominal pain, signs of peritoneal irritation, and abrupt deterioration with maternal shock	Emergent laparotomy and multiple hemostatic sutures	EBL at 1500 ml The patient recovered smoothly Stillbirth
The present case 1	21–22 weeks	Ruptured vessels and laceration on the serosal surface of the uterus	Abnormal vascular proliferation of adenomyosis and endometriosis	Abdominal tenderness, shoulder pain, hypovolemic shock Dropped Hb level US revealed a large amount of free fluid in abdominal cavity	Exploratory laparotomy and hemostasis	EBL at 2000 ml 4 units of packed RBC Maternal survival with uterine conservation Very preterm birth on the 4th postoperative day
The present case 2	34 weeks 3 days	Spontaneous rupture of vessels eroding into utero-ovarian plexus	Abnormal proliferation of utero-ovarian plexus and adhesions from previously ovarian tumor resection	Abdominal pain Low Hb levels US showed free fluid collection in abdominal cavity extended to hepatic and renal space	Exploratory laparotomy, CS, releasing adhesion, vessel ligation and hemostasis	EBL at 1200 ml 2 units of packed RBC The patient was alive and the uterus was preserved Preterm delivery with a live newborn

*CS *caesarean section,* CT *computed tomography,* EBL *estimated blood loss,* IOL *induction of labor, *GA *gestational age,* Hb *hemoglobin,* Hct *hematocrit,* FFP* fresh frozen plasma,* RBCs *red blood cells,* US* ultrasound

## Conclusion

In summary, the etiologies of SHiP are rarely established with the spontaneous rupture of proliferative vasculature relates to some unknown risk factors such as endometriosis, adenomyosis and a relevant history of ovarian tumor removal. However, SHiP in pregnancy carries a poor prognosis for both mother and fetus. Therefore, a high index of suspicion, a prompt diagnosis, and a rapid intervention are important keys to achieve favorable outcomes of unprovoked intraperitoneal bleeding.

### Supplementary Information

**Additional file 1:** **Supplementary video. 1** A-B Hemostatic procedures during emergent laparotomy of the present case 2. Video shows the resection of adherent structure, the bleeding point, and hemostatic procedures were carefully performed at surgery. 

**Additional file 2.**

## Data Availability

The datasets used and/or analyzed during the current study are available from the corresponding author on reasonable request.

## Abbreviations

CS: Caesarean section
Hb: Hemoglobin
SHiP: Spontaneous hemoperitoneum in pregnancy
GA: Gestational age
US: Ultrasound
