# Correction: Spontaneous hemoperitoneum in the second and third trimester of pregnancy: two uncommon case reports at Tu Du Hospital, in Vietnam and a literature review

**DOI:** 10.1186/s12245-023-00535-8

**Published:** 2023-09-05

**Authors:** Anh Dinh Bao Vuong, Thanh Hai Pham, Xuan Trang Nguyen, Ngoc Bich Trinh, Phuc Nhon Nguyen, Quang Nhat Ho

**Affiliations:** 1 Department of High-Risk Pregnancy, Tu Du Hospital, Ho Chi Minh City, Vietnam; 2 Tu Du Clinical Research Unit (TD-CRU), Ho Chi Minh City, Vietnam; 3 Department of Postoperative Care, Block A, Tu Du Hospital, Ho Chi Minh City, Vietnam

**Correction: Int J Emerg Med 16, 26 (2023)**

**https://doi.org/10.1186/s12245-023-00498-w**

In the original publication of Vuong et al. [1], Figs. 1F and 2C were missing due to a typesetting error. In addition, the author group identified an error in Fig. 2D. The correct figures are given below.

**Fig. 1:**
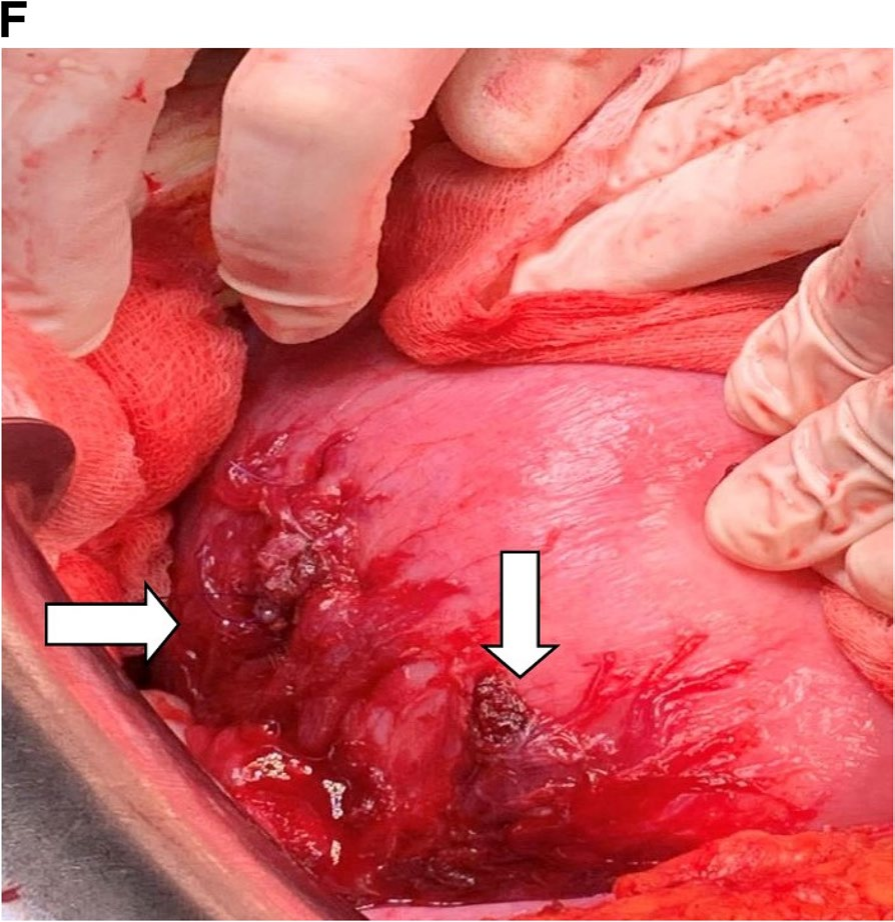
Ultrasound scan shows as follows: **A** free fluid in the pelvic cavity. **B** Single alive fetus at 21 weeks and 3 days of gestation and maternal hydronephrosis at the third grade. **C** Adenomyosis image. **D** Endometriotic cyst. **E** Hypervascularity on the lateral wall of the uterus. **F** Abnormal appearance with laceration on the serosal surface of the uterus and vessel ligations (white arrow) were performed during exploratory laparotomy

**Fig. 2:**
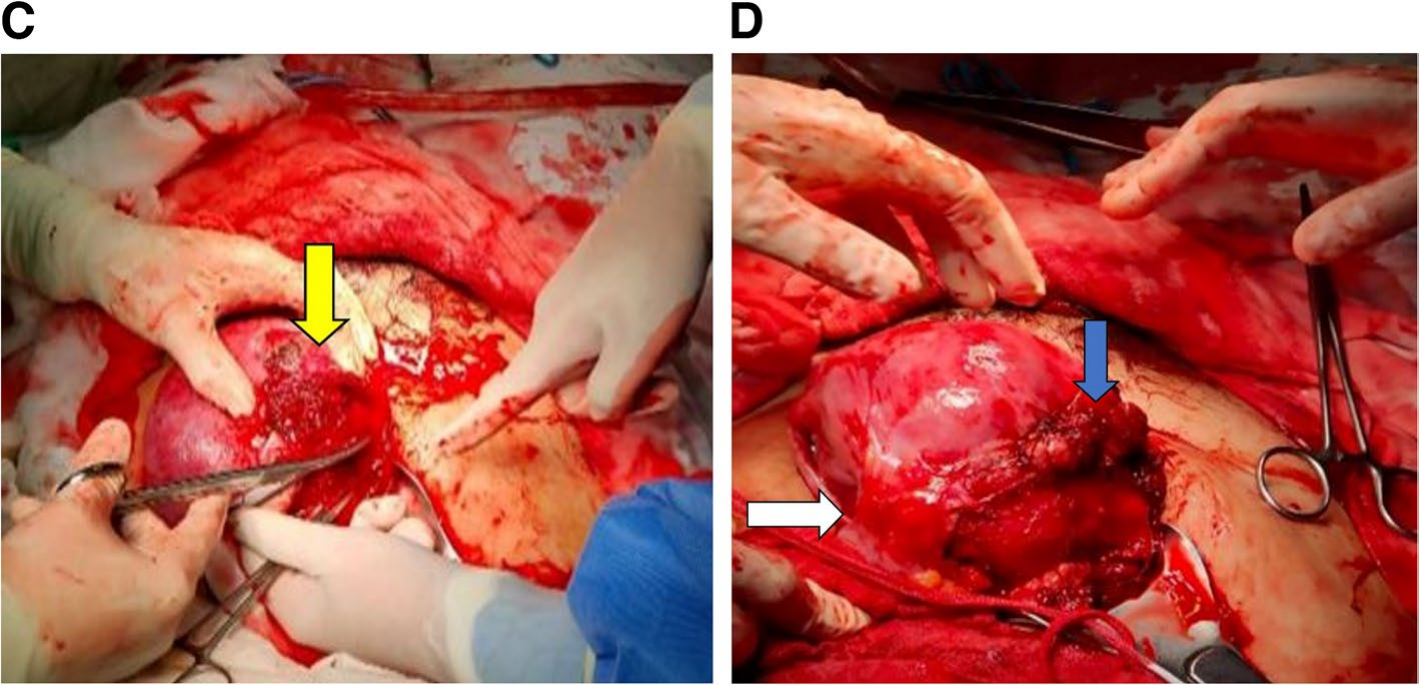
Ultrasonography shows **A** a single a live fetus and proliferative vasculature at the cervix. **B** Free fluid collection in the abdominal cavity. **C** Extravasation in the right adnexa, suspected to arise from the right utero-ovarian plexus (yellow arrow). **D** Uterine closure accompanied with a multiple hemostatic sutures were performed (blue arrow). One of the adherent bandages existed between the lateral posterior of the uterus and the abdominal anterior wall of the abdomen (white arrow). The bleeding stopped after releasing a part of the adhesion, excision of fragile tissue, and suturing

The original article [1] has been corrected.
